# Knowledge and perception of pulmonary tuberculosis in pastoral communities in the middle and Lower Awash Valley of Afar region, Ethiopia

**DOI:** 10.1186/1471-2458-10-187

**Published:** 2010-04-12

**Authors:** Mengistu Legesse, Gobena Ameni, Gezahegne Mamo, Girmay Medhin, Dawit Shawel, Gunnar Bjune, Fekadu Abebe

**Affiliations:** 1 Aklilu Lemma Institute of Pathobiology, Addis Ababa University, Addis Ababa, Ethiopia; 2 Faculty of Veterinary Medicine, Addis Ababa University, Addis Ababa, Ethiopia; 3 Department of General Practice and Community Medicine, University of Oslo, Oslo, Norway; 4 Norwegian Center for Minority Health Research, Oslo, Norway

## Abstract

**Background:**

Afar pastoralists live in the northeast of Ethiopia, confined to the most arid part of the country, where there is least access to educational, health and other social services. Tuberculosis (TB) is one of the major public health problems in Afar region. Lack of knowledge about TB could affect the health-seeking behaviour of patients and sustain the transmission of the disease within the community. In this study, we assessed the knowledge and perception of apparently healthy individuals about pulmonary tuberculosis (PTB) in pastoral communities of Afar.

**Methods:**

Between March and May 2009, a community-based cross-sectional questionnaire survey involving 818 randomly selected healthy individuals was conducted in pastoral communities of Afar region. Moreover, two focus group discussions (FGDs), one with men and one with women, were conducted in each of the study area to supplement the quantitative study.

**Results:**

The majority (95.6%) of the interviewees reported that they have heard about PTB (known locally as "Labadore"). However, the participants associated the cause of PTB with exposure to cold air (45.9%), starvation (38%), dust (21.8%) or smoking/chewing Khat (*Catha edulis*) (16.4%). The discussants also suggested these same factors as the cause of PTB. All the discussants and the majority (74.3%) of the interviewees reported that persistent cough as the main symptom of PTB. About 87.7% of the interviewees and all the discussants suggested that PTB is treatable with modern drugs. All the discussants and the majority (95%) of the interviewees mentioned that the disease can be transmitted from a patient to another person. Socio-cultural practices, e.g. sharing cups (87.6%), and house type (59.8%) were suggested as risk factors for exposure to PTB in the study areas, while shortage of food (69.7%) and chewing khat (53.8%) were mentioned as factors favouring disease development. Almost all discussants and a considerable number (20.4%) of the interviewees thought that men were the highest risk group to get PTB as well as playing a major role in the epidemiology of the disease.

**Conclusion:**

The findings indicate that pastoral communities had basic awareness about the disease. Nevertheless, health education to transform their traditional beliefs and perceptions about the disease to biomedical knowledge is crucial.

## Background

Pastoralism accounts for the livelihoods of 50-100 million people in developing countries, while ~60% of this population live in more than 21 African countries confined to the most arid regions of the continent [1,2]. In East Africa, Ethiopia has the largest pastoralist population (7-8 million), and the majority is found in Afar region [3]. Afar pastoralists depend on livestock for their livelihood, moving seasonally from place to place with their animals in search of water and pasture. Hence, they have the least access to educational, health and other services. The Afar pastoralists are a distinct ethnic group, with their own culture and language [4].

Tuberculosis (TB) is one of the major diseases that cause enormous public health and economic crisis in low income countries [5]. Factors such as HIV/AIDS, smoking and malnutrition have been identified as substantial contributors to the epidemiological burden of active TB [5-8]. However, the risk factors for exposure to TB are different from the risk factors for disease development. Poverty and lack of awareness are considered the most important factors that increase the risk of exposure to TB [9,10]. Lack of knowledge about the cause, mode of transmission, and symptoms, as well as appropriate treatment of TB not only affect the health-seeking behaviour of patients, but also could affect control strategy, thereby sustaining the transmission of the disease within the community [11-14]. For these reasons, creating general awareness about TB among communities and initiating community participation in the control of the disease make up 1 component of the 6 basic components of the "Stop TB Strategy" of the World Health Organization (WHO) [15].

According to the WHO 2009 report on the epidemiological burden of TB, Ethiopia is ranked 7^th ^among the 22 countries in the world with a high-burden of TB [5]. The disease is also one of the major public health problems in Afar pastoralists [16], and the region is ranked 2^nd ^with a notification rate between 146 - 260 per 100, 000 population within the country [5]. To the best of our knowledge, there is no reliable information on the prevalence, incidence or community's perception and knowledge of the disease in the region. As part of a large on-going study on TB in Afar pastoralists and their livestock, we conducted a questionnaire survey to explore what the pastoral communities know about the cause, mode of transmission, symptoms, prevention and treatment of PTB.

## Methods

### Study Area and Population

The study was conducted in Dubti and Amibara Districts, Afar region, North-East Ethiopia. The region has a total population of 1,411,092 with an estimated area of 96,707 square kilometers [17]. In the region, population density is about 14.6 persons/sq km though it varies from zone to zone. According to Medicin Sans Frontieres report (16) TB is the leading cause of morbidity in the region. Dubti District is in the Lower Awash valley, approximately at 574 km to the North-East of Addis Ababa. It has 18 small administrative units (kebeles) of which 3 are towns. The district has ~87,000 population of whom 27.8% are urban dwellers [18]. Amibara District is found in the Middle Awash valley ~260 km to the East of Addis Ababa. It has 18 kebeles of which 4 are towns/semi-towns. The district has ~54,000 population of whom 52.4% are urban dwellers [18]. The majority of the pastoral population of the 2 districts is nomadic, while some of them are practicing agro-pastoralism. Pastoralists of Dubti District migrate to various other districts during dry season, while Amibara's pastoralists migrate within the district.

The 2 districts were conveniently selected for a major study of the prevalence of latent and active TB in pastoralists and their livestock. However, prior to the implementation of a survey on the prevalence of the disease, we attempted to assess the knowledge and perception of the communities about PTB. There was no previous information on the level of pastoral community awareness about PTB in the present study areas or in the region as whole. Thus, based on the assumption that 50% of the participants in the study districts had high knowledge of PTB, (95% confidence and 5% degree of accuracy) and a design effect of 1.1 due to multi-stage sampling, a total of 424 participants were included from each of the selected districts. The participants were eligible if they were the member of that kebele, a husband/wife (or the responsible person) in the selected households, apparently healthy and willing to volunteer to be interviewed. The study protocol was approved by the Ethical Clearance Committee of the Aklilu Lemma Institute of Pathobiology (ALIPB), Addis Ababa University as well as by the Regional Committee for Medical Research Ethics of Southern Norway. The aim of the study was explained to each of the participant and verbal consent was obtained. Each participant was interviewed independently and the collected information was kept confidential. In case of refusal, it was planned to interview a person from the next household.

### Study Design and Data Collection

Between March and May 2009, a community-based cross-sectional survey was conducted in randomly selected pastoralists' kebeles of the 2 districts. Prior to data collection, a list of all the kebeles in the selected districts was obtained from the respective District Health Office. Based on this list, 7 and 6 pastoral kebeles were selected from Amibara and Dubti districts, respectively. The selected kebeles were stratified into manageable villages and a list of households of each village was prepared. Based on the number of households in each kebele, the pre-estimated sample size (424) was proportionally distributed. The required number of participants (husband or wife) was selected using systematic random sampling from each kebele using these lists.

Structured and some open-ended questionnaires were prepared in English, based on information from available literature [19-21]. The questionnaires were translated into Amharic and pre-tested for clarity and cultural acceptability in the districts. During pre-testing, additional information was gathered and some of the questionnaires were modified. The participants were interviewed in their local language by trained data collectors (diploma graduate elementary school teachers) selected from the localities. Each interview was made by a house-to-house visit. Information on the socio-demographic characteristics of the participants was also included in the questionnaires.

After completing the quantitative data collection, 2 FGDs (one with men and one with women) comprised of 8-10 men or women who were not involved in the individual interview were conducted in Hanekisna-Arado Kebele, Dubti District. Similarly, 2 FGDs (one with men and one with women) were conducted in Angellele Kebele, Amibara District. These 2 kebeles were selected by a lottery system among the kebeles selected for the quantitative data collection. The discussion was made with men and women separately, at different times on the same day. Specific topics were prepared as guides for the discussion, moderated by the principal investigator and a trained health worker. The topics were presented one by one, allowing adequate discussion on each topic. The response was recorded using a notebook, translated into Amharic and then into English. Socio-demographic characteristics of the participants were recorded during the discussion

### Data Analysis

The collected data were re-translated to English, coded and double-entered into a data entry file using EpiData software, V.3.1. The data were transferred to SPSS software V.16 and analyzed according to the different variables. Pearson chi-square was used to evaluate the statistical significant of bivariate association of gender and selected covariate in each district. Bivariate and multivariable logistic regression analysis was performed to explore independent variables that were predictors of overall knowledge of PTB as well as that of the four subscales of knowledge of PTB (sign/symptoms, mode of transmission, knowledge of effective treatment and preventive methods) [21,22]. Differences were considered significant when p < 0.05.

Overall knowledge of the study participants about PTB was assessed using the following 8 main questions: (1) able to mention bacteria/germ as a cause of PTB, (2) able to mention correct sign/symptoms of PTB (persistent cough for three or more weeks, sputum with blood, chest pain, weight loss, loss of appetite and fever/sweat), (3) able to classify PTB as a transmissible disease, (4) able to enumerate correct mode of transmission of PTB (cough/breath, sharing cups, not sharing feeding materials, not through body contact or sharing cloths), (5) knowing that PTB is treatable, (6) knowing that effective treatment for PTB is modern drug, (7) knowing that PTB is preventable, and (8) able to enumerate correct preventive methods of PTB (avoiding sharing cups, using separate room, early treatment). Response to these questions were added together to generate a knowledge score ranging from 0 to 18. After assessing normality to the score using histogram, the composite score was dichotomized using mean as a cut-off value so that higher value coded as 1 showing higher overall knowledge of PTB in this community

A score of one was given to correct responses, zero being used for incorrect/do not know responses. Based on the mean score of the composite variable (mean = 10.06), the responses were categorised into high (score above mean value) and low (score below mean value) knowledge of PTB. Similarly, scores were generated for the four subscales of knowledge of PTB and categorized into high and low knowledge of each domain using mean value.

Information collected during the FGDs was translated into Amharic and entered into separate tables for women and men, according to the study area. Responses that reflected the common views of the discussants were selected, translated into English. The accuracy of the translation was checked by re-translating into Amharic and then into the local language (Afargna) by another person. Responses from each discussant were compared for similarities/differences and analyzed using content method [23].

## Results

### Socio-demographic characteristics

A total of 818 participants (age range 18-70, mean age 36.9 years) involved in the study from the 2 areas. Of this figure 394 (48.2%) participants were from the Dubti District, while 424 (51.8%) were from the Amibara District. The majority of the participants were pastoralists (71.6%), most being illiterate (92.1%) (Table 1).

**Table 1:** Socio-demographic characteristics of the participants

Characteristic	Dubti; Number (%)	Amibara; Number (%)	Total (%)
Gender:			
Female	162 (41.1)	195 (46.0)	357 (43.6)
Male	232 (58.9)	229 (54.0)	461 (56.4)
Age (years):			
18-29	68 (17.3)	140 (33.0)	208 (25.4)
30-44	227 (57.6)	190 (44.8)	417 (51.0)
45-59	87 (22.1)	65 (15.3)	152 (18.6)
60+	12 (3.0)	29(6.8)	41 (5.0)
Martial status:			
Married	375 (95.7)	392 (92.5)	767 (94.0)
Other	17(4.3)	32 (7.5)	51 (6.0)
Ethnicity:			
Afar	394 (100)	423 (99.8)	817 (99.9)
Other	0 (0)	1(0.2)	1 (0.1)
Region:			
Muslim	394 (100)	424 (100)	818 (100)
Occupation:			
Pastoralist	260 (66.0)*	326 (76.9)*	586 (71.6)
Agro-pastoralist	134 (34.0)	98 (23.1)	232 (28.4)

Educational status:			
Illiterate	361(91.6)	392 (92.5)	753 (92.1)
Primary	19 (4.8)	11(2.6)	30 (3.7)
Secondary	4 (1.0)	2 (0.5)	6 (0.7)
Other (read & write)	9 (2.3)	20 (4.7)	29 (3.5)

### Communities' Knowledge of the Cause, Symptoms and Treatment of PTB

Out of the 818 participants, 782 (95.6%) reported that they had heard about PTB (known locally as "Labadore") mainly from friends or PTB patients. However, only 2 participants mentioned that bacteria/germs were the cause of PTB. Cold air (45.9%), shortage of food (38.0%), dust (21.8%) and smoking/chewing khat (*Catha edulis*) (16.4%) were the frequently mentioned factors as the cause of the disease. Table 2 shows the communities' knowledge about cause, symptoms and treatment of PTB. A higher proportion of participants from the Dubti area suggested shortage of food as the cause of the disease compared to participants from the Amibara area (60.6% vs 19.5%, p < 0.001). A larger proportion of participants from the Dubti area mentioned persistent cough as a major symptom of PTB than participants from the Amibara (78.9% vs 70.1%, p = 0.005). The majority (94.2%) of the participants from both areas knew that PTB is treatable with modern drugs (87.7%). Moreover, 215 (27.5%) participants (Table 3) reported that either themselves or their families had previously got PTB and were treated with these drugs at health facilities. Herbal treatment (72.2%) was frequently mentioned by individuals who suggested traditional treatment, while others mentioned camel's milk and goat's meat as remedies.

**Table 2:** Communities' knowledge about cause, symptoms and treatment of PTB

Variable	Dubti			Amibara			
	**Male (%)**	**Female (%)**	**Total (%)**	**Male (%)**	**Female (%)**	**Total (%)**	**Total (%)**

Cause of PTB:							
Bacteria/germ	1(0.5)	0 (0)	1(0.3)	1 (0.5)	0 (0)	1 (0.3)	2 (0.3)
Cold air	84 (41.8)	66 (52.0)	150 (45.7)	97 (44.7)	86 (47.3)	183 (45.9)	333 (45.9)
Shortage of food	123 (61.2)	75 (59.7)	198 (60.6)*	35 (16.1)	43 (23.6)	78 (19.5)*	276 (38.0)
Smoking/chewing	25 (12.4)	16 (7.4)	41(12.5)*	39 (18.0)	39 (21.4)	78 (19.5)*	119 (16.4)
Sun light	32 (15.9)	17 (13.5)	49 (15.0)*	16 (7.4)	19 (10.4)	35 (8.8)*	84 (11.6)
Dust	33 (16.4)	29 (23.0)	62 (19.0)	46 (21.2)	50 (27.5)	96 (24.1)	158 (21.8)
Do not know	38 (18.9)	17 (13.5)	55 (16.8)*	46 (21.2)	52 (28.6)	98 (24.6)*	153 (21.1)
Symptoms of PTB:							
Cough for 3 weeks	176 (79.6)	116 (77.9)	292 (78.9)*	154 (69.4)	132 (71.0)	286 (70.1)*	578 (74.3)
Sputum with blood	79 (35.7)	61(40.9)	140 (37.8)*	123 (55.4)	107 (57.5)	230 (56.4)*	370 (47.6)
Weight loss	85 (38.5)	55 (36.9)	140 (37.8)	88 (39.6)	62 (33.3)	150 (36.8)	290 (37.3)
Loss of appetite	61(27.6)	45 (30.2)	106 (28.6)*	37(16.7)	45 (24.2)	82 (20.1)*	188 (24.2)
Fever & sweat	61(27.6)	49 (32.9)	110 (29.7)*	34 (15.3)	38 (20.4)	72 (17.6)*	182 (23.4)
Chest pain	31(14.0)	28 (18.8)	59 (15.9)*	66 (29.7)	64 (34.4)	130 (31.9)*	189 (24.3)
Do not know	2 (0.9)	6 (4.0)	8 (2.2)*	12 (5.4)	29 (15.6)	41 (10.1)*	49 (6.3)
PTB is treatable:							
Yes	217 (98.2)	146 (97.3)	363 (97.8)*	204 (91.9)	166 (89.7)	370 (90.9)*	733 (94.2)
No	1 (0.5)	4 (2.7)	5 (1.3)	6 (2.7)	4 (2.2)	10 (2.5)	15 (1.9)
Do not know	3 (1.4)	0 (0)	3 (0.8)	12 (5.4)	15 (8.1)	27 (6.6)	30 (3.9)

PTB treatment							
Modern drug	196 (90.3)*	119 (81.5)*	315 (86.8)	187 (91.7)	141 (84.9)*	328 (88.6)	643 (87.7)
Traditional medicine	17 (7.8)	25(17.1)	42 (11.6)	17 (8.3)	25 (15.1)	42 (11.4)	84 (11.5)
Both	4 (1.8)	2 (1.4)	6 (1.7)	-	-	-	6 (0.8)

* significant difference between male and female, or between participants from the two study areas (P < 0.05)

**Table 3:** Communities' perception about public health importance of PTB

Variable	Dubti			Amibara			
	**Male (%)**	**Female (%)**	**Total (%)**	**Male (%)**	**Female (%)**	**Total (%)**	**Total (%)**

PTB is a public health problem in your area:							
Yes	109 (49.3)	77 (51.3)	186 (50.1)*	53 (23.5)	57 (29.8)	110 (26.4)*	296 (37.6)
Rare	104 (47.1)	66 (44.0)	170 (45.8)	151 (66.8)	102 (53.4)	253 (60.7)	423 (53.7)
No	2 (0.9)	2 (1.3)	4 (1.1)	22 (9.7)	26 (13.6)	48 (11.5)	52 (6.6)
Do not know Since when?	6 (2.7)	5 (3.3)	11(3.0)	0 (0)	6 (3.1)	6 (1.4)	17 (2.2)
Since many years ago	123 (57.7)	87 (60.8)	210(59.0)*	174 (85.3)	143 (89.4)	317 (87.1)*	527 (73.2)
Since recent years	90 (42.3)	56 (39.2)	146 (41.0)*	30 (14.7)	17 (10.6)	47(12.9)*	193 (26.8)
Do not know	0 (0)	0 (0)	0 (0)	0 (0)	0 (0)	0 (0)	0 (0)
Family sick from PTB:							
Yes	59 (26.7)	47 (31.3)	106 (28.6)	51 (23.0)	58 (31.4)	109 (26.8)	215 (27.6)
No	162 (73.3)	103 (68.7)	265 (71.4)	171 (77.0)	127 (68.6)	298 (73.2)	563 (72.4)
PTB mostly attacks:							
Male	52 (23.6)*	21 (14.0)*	73 (19.7)	57 (25.7)*	28 (15.2)*	85 (20.9)	158 (20.4)
Female	23 (10.5)	6 (4.0)	29 (7.8)	9 (4.1)	4 (2.2)	13 (3.2)	42 (5.4)
Both	135 (61.4)	113 (75.3)	248 (67.0)	130 (58.6)	112 (60.9)	242 (59.6)	490 (63.1)
Do not know	10 (4.5)	10 (6.7)	20 (5.4)	26 (11.7)	40 (21.7)	66 (16.3)	86 (11.1)

PTB mostly attacks:							
Under 5 years	145 (65.6)	103 (68.7)	248 (66.8)*	97 (43.7)	74 (40.0)	171 (42.0)*	419 (53.9)
Five-fifteen years	161 (73.2)	118 (78.7)	279 (75.4)*	100 (45.0)	93 (50.3)	193 (47.4)*	472 (60.7)
Adult under 60 years	157 (71.0)	108 (72.0)	265 (71.4)	151 (68.0)	130 (70.3)	281 (69.0)	546 (70.2)
Over 60 years	186 (84.2)	116 (77.3)	302 (81.4)	168 (75.7)	146 (78.9)	314 (77.1)	616 (79.2)
Do not know	2 (0.9)	5 (3.3)	7 (1.9)	19 (8.6)	23 (12.4)	42 (10.3)	49 (6.3)

* significant difference between male and female, or between participants from the two study areas

### Communities' Knowledge of the Mode of Transmission and Prevention of PTB

Table 4 depicts the communities' knowledge about the mode of transmission and preventive methods of PTB. The majority (95%) of the participants from both the study areas knew that PTB can be transmitted from a patient to another person. Relatively, a higher proportion (97.8% vs 92.3%, p = 0.001) of participants from Dubti mentioned that PTB is a transmissible disease compared to participants from Amibara. A higher proportion (95.4% vs 88.7%, p = 0.011) of men in Amibara mentioned that PTB is a transmissible disease compared to women from the same area. Cough/breath (80.8%) and sharing cups (77.6%) with a patient were the most frequently mentioned routes of transmission by participants from both areas. Others also mentioned that the disease can be transmitted through other routes, including sharing tooth brushes, cigarettes, or sexual intercourse. The majority (82.5%) of the participants from both areas responded that transmission of PTB would be preventable mainly by avoiding sharing cups (94%) with a patient, and using separate rooms (70.5%). Abstinence from sex, early treatment, avoidance of avoiding spiting everywhere, and personal hygiene were also mentioned by some of the participants as methods of preventing the disease.

**Table 4:** Communities' knowledge of mode of transmission and prevention of PTB

Variable	Dubti			Amibara			
	**Male (%)**	**Female (%)**	**Total (%)**	**Male (%)**	**Female (%)**	**Total (%)**	**Total (%)**

PTB can be transmitted:							
Yes	217 (98.6)	143 (96.6)	360 (97.8)*	209 (95.4)	165 (88.7)*	374 (92.3)*	734 (95.0)
No	2 (0.9)	4 (2.7)	6 (1.6)	2 (0.9)	11(5.9)	13 (3.2)	19 (2.5)
Do not know	1 (0.5)	1(0.7)	2 (0.5)	8 (3.7)	10 (5.4)	18(4.4)	20 (2.6)
PTB transmitted through:							
Cough/breath	182 (83.9)	111(76.6)	293 (80.9)	175 (82.5)	128 (78.1)	303 (80.6)	596 (80.8)
Sharing cups	153 (70.5)	112 (77.2)	265 (73.2)*	174 (82.1)	134 (81.7)	308 (81.9)*	573 (77.6)
Sharing feeding materials	86 (39.6)	60 (41.4)	146 (40.3)*	109 (51.4)	95 (57.9)	204 (54.3)*	350 (47.4)
Other (sex, contact, fly)	23 (10.6)	12 (8.3)	35 (9.7)	8 (3.8)	4 (2.4)	12 (3.2)	47 (6.4)
Do not know	0 (0.0)	1 (0.7)	1 (0.3)	1 (0.5)	5 (3.1)	6 (1.6)	7 (0.9)
PTB is preventable:							
Yes	186 (84.9)	118 (79.2)	304 (82.6)	192 (87.3)	141 (76.6)*	333 (82.4)	637 (82.5)
No	15 (6.8)	12 (8.1)	27 (7.3)	7 (3.2)	11(5.9)	18 (4.5)	45 (5.8)
Do not know	18 (8.2)	19 (12.8)	37 (10.1)	21(9.5)	32 (17.4)	53 (13.1)	90 (11.7)

Preventive methods:							
Avoiding sharing cups	173 (93.0)	108 (92.3)	281 (92.7)	180 (93.8)	138 (97.2)	318 (95.2)	599 (94.0)
Using separate room	131 (70.4)	89 (76.1)	220 (72.6)	129 (67.2)	100 (70.4)	229 (68.6)	449 (70.5)
Other (early treatment, food, avoid sex,)	21(9.6)	11(7.4)	32 (8.7)	9 (4.1)	2 (1.1)	11(2.7)	43 (5.6)
Do not know	1 (0.5)	1 (0.9)	2 (0.7)	5 (2.6)	2 (1.4)	7 (2.1)	9 (1.4)

* significant difference between male and female, or between participants from the two study areas (P < 0.05)

Among 215 participants who reported a previous history of PTB (Table 3), 160 (74.4%), 192 (89.3%) and 29 (13.5%) reported that they used separate sleeping places, separate utensils and other methods (early treatment or avoiding spitting everywhere) to prevent transmission of the disease to other family members, respectively.

### Communities' Perception of Socio-Cultural Risk Factors for Exposure to PTB

Most of the participants from both the study areas suggested that the habit of sharing a single cup among several individuals (87.6%) and the type of house (locally known as an Afar house) (59.8%) were the major socio-cultural risk factors for exposure to PTB (Table 5). More than half of the participants believed that food scarcity (69.7%) and the frequent chewing of khat (53.8%) were risk factors for disease development. A higher proportion of the participants from Dubti associated lack of food with the risk of disease development than from Amibara (87.5% vs 53.5%, p < 0.001).

**Table 5:** Risk factors for exposure to PTB and disease development

Variable	Dubti			Amibara			
	**Male (%)**	**Female (%)**	**Total (%)**	**Male (%)**	**Female (%)**	**Total (%)**	**Total (%)**

Risk factors for exposure:							
Cups sharing habit	195 (89.0)	132 (88.6)	327 (88.9)	191 (88.8)	147 (83.5)	338 (86.4)	665 (87.6)
House type	155 (70.8)	87 (58.4)	242 (65.8)*	114 (53.0)	98 (55.7)	212 (54.2)*	454 (59.8)
Chewing khat together	86 (39.3)	53 (35.6)	139 (37.8)	98 (45.6)	69 (39.2)	167 (42.7)	306 (40.3)
Other (sleeping with patient, spitting everywhere)	20 (9.1)	10 (6.7)	30 (8.2)	2 (0.9)	0 (0)	2 (0.5)	32 (4.2)
Do not know	9 (4.1)	7 (4.7)	16 (4.4)	14 (6.5)	21(11.9)	35 (8.9)	51 (6.7)

Risk factors for disease:							
Shortage of food	192 (88.1)	123 (86.6)	315 (87.5)*	126 (58.1)	86 (48.0)	212 (53.5)*	527 (69.7)
Chewing & smoking	133 (61.0)	79 (55.2)	212 (58.7)*	106 (48.8)	89 (49.7)	195 (49.2)*	407 (53.8)
Stress	63 (28.9)	31(21.7)	94 (26.0)*	34 (15.7)	39 (21.8)	73 (18.4)*	167 (22.1)
Other chronic disease	35 (16.1)	22 (15.5)	57 (15.9)	25 (11.5)	26 (14.5)	51(12.9)	108 (14.3)
Other (sex, work load)	14 (6.4)	12 (8.5)	26 (7.2)*	34 (15.7)	24 (13.4)	58 (14.6)*	84 (11.1)
Do not know	8 (3.7)	6 (4.2)	14 (3.9)*	25 (11.5)	45 (24.9)	70 (17.6)*	84 (11.1)

* significant difference between male and female, or between participants from the two study areas (P < 0.05)

### Perception of Communities about Public Health Importance of PTB

Table 3 shows the communities' perception about the importance of public health of PTB. A higher proportion of participants from Dubti considered PTB as a major public health problem in their area than participants from Amibara (50.1% vs 26.4%, p < 0.001). A higher proportion of participants from Dubti indicated that PTB is becoming a major public health problem in recent years compared to those from Amibara (41.0% vs 12.9%, p < 0.001). Among individuals who believed there had been a recent expansion of the disease, the majority (86.0%) associating it with a shortage of food in the area. Some, however, mentioned smoking/chewing khat (30.6%), climate change (16.6%), HIV/AIDS (1%) and other factors (water problems, work-load or population increase) as responsible factors (16.1%). A considerable number of participants (20.4% vs 5.4%, p < 0.001) believed that men are more frequently attacked by PTB than women; and most participants from both the study areas thought that PTB mostly attacks persons older than 60 years (79.2%).

Crude and adjusted effects of selected covariates obtained from logistic regression are summarized in Table 6 for the overall knowledge. Similarly, crude and adjusted effects of selected covariates obtained from logistic regression are presented in additional file for the four sub-domains of overall knowledge of PTB (Additional file 1: Table S1). High knowledge of the choice of modern drugs as effective treatment for PTB was significantly associated with men (adjusted OR, 2.21; 95%CI, 1.37 to 3.57; p = 0.001). Better knowledge of identifying symptom (adjusted OR, 3.66 95%CI, 2.63 to 5.08; p < 0.001) and identifying preventive methods of PTB (adjusted OR, 3.79; 95%CI, 2.42 to 5.93; p < 0.001) were significantly associated with agro-pastoralism as an occupation (additional file 2). On the other hand, agro-pastoralism as an occupation (adjusted OR, 7.85; 95% CI, 5.07 to 12.14; p <0.001) and age between 45 and 59 years (adjusted OR, 1.91; 95% CI, 1.05 to 3.49; p = 0.035) were significantly associated with high overall knowledge of PTB (Table 6).

**Table 6:** Association of respondents' socio-demographic characteristics with respondents' overall knowledge of PTB

Characteristic	Crude OR(95%, CI)	Adjusted OR(95%, CI)
District		
Dubti	Reference	Reference
Amibara	1.28 (0.91- 1.82)	1.47(0.98 - 2.19)
Gender:		
Female	Reference	Reference
Male	0.77 (0.54- 1.10)	0.75 (0.49- 1.13)
Age (years):		
18-29	Reference	Reference
30-44	1.02 (0.66 - 1.56)	0.96 (0.59 - 1.57)
45-59	1.96 (1.16 - 3.30)	1.91(1.05 - 3.49)
60+	1.06 (0.44 - 2.55)	0.92 (0.34 - 2.49)
Educational status		
Illiterate	Reference	Reference
Literate	1.15 (0.63 - 2.11)	1.46 (0.72 - 2.95)

Occupation:		
Pastoralist	Reference	Reference
Agro-pastoralist	7.7 (5.05 - 11.87)	7.85 (5.07 - 12.14)

### Focus Group Discussion

A total of 18 participants (10 men and 8 women, age range 24-70, mean age 40.7 years) involved in the FGDs held at the Hanekisna-Arado kebele, whereas, 20 participants (10 men and 10 men, age range from 20-80, mean 43.9 years) involved at the Angellele kebele. Among the 18 participants from Hanekisna-Arado, 13 (72.2%), 5(27.8%) and 18(100%) were pastoralists, agro-pastoralists and illiterate, respectively. Out of the 20 participants from Angellele, the corresponding figures were 15 (75%), 5 (25%) and 18 (90%).

According to men and women discussants from Hanekisna-Arado, PTB was the most important public health problem, followed by skin disease and malaria. Men and women discussants from Angellele placed PTB as third position, next to diarrhoea and urinary schistosomiasis. The participants from both kebeles suggested that dust, shortage of food, chewing khat/smoking and cold air were causes of PTB. Most of the men and women discussants from both kebeles believed that dust deposits in the lung can result in PTB. But, a male participant from Hanekisna-Arado said that "*If dust could cause PTB, all persons who are involved in lorry driving and road construction would suffer from it*." A 70-year old man from the same kebele said that *"I was the victim of PTB. I used to smoke and chew khat frequently and eventually I got the disease because of this habit. I believe that the cause of this disease is frequent chewing khat and smoking*."

All discussants from both kebeles mentioned persistent cough and sputum with blood as the main symptoms of PTB, while modern drugs were suggested as the effective treatment. The discussants mentioned that using a separate room for a patient is a good way of preventing transmission of the disease. All of the discussants from both areas mentioned that living with a PTB patient in a small house like a Afar home and the habit of sharing cups were the major risk factors for exposure to the disease. Almost all discussants from both kebeles thought that men were the highest risk group of PTB. Because of 1) men usually chew khat, 2) share cigarette and cups for drinking water during chewing, 3) move from place to place for various purposes (e.g. following livestock), they share utensils and are exposed to dust.

The discussants from both kebeles suggested that lack of food as the main risk factor for developing the disease. A male discussant from Angellele stated that "*someone can be exposed to either a dust or acquired the disease from mother's or cow's milk during childhood, but became a patient later when he/she lacks resistant due to age or shortage of food"*. The discussants from both kebeles mentioned that PTB has becoming a major public health problem in recent years because of poverty, climate change and migration of daily labours to the areas from other parts of Ethiopia. The participants also strongly complained that delay in treatment is one of the major factors contributing to the expansion of the disease, as most patients do not visit health facilities as soon as they get sick.

## Discussion

The results of this study indicated that PTB is familiar to the pastoral communities in the present study areas, as the majority (95.6%) of the participants reported that they have heard about PTB ("Labadore") mainly from friends or PTB patients. Moreover, the discussants from both the study areas indicated that PTB is one of the most important public health problems of the present study areas. Nevertheless, similar to the findings of community based studies from other parts of Ethiopia [19,22] as well as from Vietnam [21], Tanzania [23] and Kenya [24], the participants had little or no information regarding the causative agent of PTB. The majority of the interviewees and discussants associated the cause of PTB mainly with either exposure to cold air, starvation, dust, or frequent smoking/chewing Khat, which is similar to the beliefs found in a previous study in another part of Ethiopia [19]. While the community perception about the role of starvation and smoking as the cause of the disease cannot be neglected [7,8,25], misconception about the correct cause of the disease could affect patient attitude towards health-seeking behaviour and preventive methods. Particularly, smoking could affect the care seeking behaviour of smokers, as the smokers may perceive their prolong cough as the cause of smoking, but not TB which could lead to delayed diagnosis and treatment.

On the other hand, the findings from this survey indicate that pastoral communities living in both of the study areas had basic awareness about the symptoms and treatment of PTB, which is comparable to the results of previous studies from this country [19,22], as also from Tanzania [26]. Pastoral community attitudes regarding treatment of the disease using modern medicine was also very high compared to the results of previous studies conducted in other parts of Ethiopia, either in communities [19,22] or in TB patients [12,13], as also seen in Tanzania [26] and Kenya [24]. TB may be perceived by a community as a non-treatable disease due to inadequate knowledge about it and appropriate treatments, which could lead to delayed diagnosis and treatment [11-14,24,27]. The high level of awareness about symptoms and appropriate treatment of PTB we observed in the studied communities could have significant implications in reducing diagnosis and treatment delay, as well as the spread of the disease.

We also noticed that pastoral communities' knowledge about the mode of transmission and preventive methods of PTB was high compared to previous findings [19,21,22,26]. However, based on the information obtained from the individual study participants knowledge of early diagnosis and treatment, which is crucial in reducing the spread of the disease, seems to be poor in the communities we have just studied. This might be due to the fact that people may not suspect that early symptoms (coughing, fever and sweating) are due to PTB, unless accompanied by other severe symptoms (e.g. chest pain or hemoptysis) [24]. On the other hand, participants in the FGDs indicated that early diagnosis and treatment is one of the main preventive methods of transmission of PTB. This implies that FGD is a powerful method of stimulating the participants and generating more crucial information than the interview method [24].

Community-based studies in South part of Ethiopia [19], Kenya [24] and Tanzania [26] showed several social-cultural factors that increase the risk of acquiring TB. In the present study, the individual participants as well as the discussants claimed that socio-cultural factors, such as living in single-room (Afar house), the tradition of sharing single cup among several individuals regardless of their healthy status could play a role in the exposure to and spread of PTB. In connection with these socio-cultural activities, a notion was prevalent both among the individual participants and the discussants that men play a major role in the epidemiology of PTB. Similar perception has been observed in community-based studies done in other countries [28,29]. In fact, the present community observation reflects the higher TB notification rates reported in men than women by WHO [5]. Among other factors, smoking and chewing of khat which are predominantly behaviour of men in the present study areas were suggested as factors associated with the high risk of acquiring PTB among men. This community concern supports the findings of study by Watkins and Plant [30] which indicated that smoking is a significant predictor of the variance in sex ratio of TB case notifications among TB high-burden countries. Hence, socio-cultural practices that appear to promote the spread of the disease should be taken into account in community-based health education pertaining to TB intervention.

HIV/AIDS is known to play a major role in increasing the burden of TB [5]. Very few individuals we studied considered the role of HIV/AIDS as a risk factor in PTB development. On the other hand, our study of communities underlined that shortage of food and the habit of using khat and smoking as major risk factors not only for the development of PTB, but also in its expansion. This is in agreement with the concern that malnutrition/micronutrient deficiency or smoking could increase the risk of developing TB [7,8]. Afar pastoralists often consume milk with local bread. In addition, the region has been facing repeated drought to the extent of causing a severe shortage of milk [31]. Thus, the present communities' perception that shortage of food as a potential risk factor in disease development and increasing the burden of PTB in the areas studied seems reasonable.

In the present study, multivariable logistic regression analysis showed that agro-pastoralism as an occupation is a predictive of high biomedical overall knowledge of PTB, which is consistent with the finding of a previous study from Eastern Ethiopia [14] and in the pastoral and agro-pastoral communities in Tanzania [26]. This might be due to the fact that nomadic pastoralists have least access to health and other social services [1,2]. This requires special attention in designing health education that fits with the nomadic mode of life, such as by selecting individuals from nomads, as well as training and recruitment as nomadic community health workers [1]. This study also revealed an association of high knowledge of choice of modern drug as effective treatment for PTB with being men participants which could have an implication on the differences in health-seeking behaviour of men and women as well as on high TB notifications among men [5].

Although the present study provides important information on the knowledge and perception of the Afar pastoral communities, it has limitations. The primary limitation is the selection of the study participants using systematic random sampling, while simple random selection method is more powerful in increasing the validity/reliability as well as reduces systematic errors and biases. Although the aim of the qualitative portion of the study was to supplement the quantitative part, the way the response was recoded and lack of detail separate discussion with pastoralist and agro-pastoralist participants could hamper generation of detail additional information. This also hindered an in-depth analysis of the results. Hence, the findings from the qualitative portion of the study might be considered preliminary.

## Conclusion

Our findings indicate that the majority of the pastoral community members in the areas we studied had a basic awareness about PTB. Nevertheless, there is a gap between their traditional knowledge and biomedical knowledge. For instance, a considerable number of the participants believed that shortage of food was the cause of PTB, as well as being the risk factor for disease development. Surprisingly, very few of them thought that having sufficient food was a preventive method of the transmission of PTB, or a treatment for the disease. Hence, health education programmes to transform their traditional beliefs and perceptions about the disease to biomedical knowledge is crucial. The results also revealed useful information on socio-cultural and occupational factors that need to be considered when designing community-based control strategies for TB.

## Competing interests

The authors declare that they have no competing interests.

## Authors' contributions

ML designed the study, participated in data collection, analysis and drafted the manuscript. GA, participated in study design, data collection, analysis and write-up. GM participated in study design, data collection and write-up. GMD, participated in study design, data analysis and interpretation. DS participated in data analysis, interpretation and write-up. GB involved in study design and critically revised the manuscript. FA involved in study design, data analysis and write-up of the manuscript and critically revised the manuscript. All authors read and approved the final manuscript. ML is the guarantor of the paper.

## Pre-publication history

The pre-publication history for this paper can be accessed here:

http://www.biomedcentral.com/1471-2458/10/187/prepub

## Supplementary Material

Additional file 1 **Table S1. Association of respondents' socio-demographic characteristics with respondents' knowledge of symptoms, mode of transmission, choice of effective treatment and preventive methods of PTB**. Association of respondents socio-demographic characteristics and four domains of the level of knowledge about PTB is investigated using logistic regression. Odds ratio and 95% CI are reported within the body of the table. Click here for file

Additional file 2 **Questionnaires administered in the study**. The questionnaire has all the questions that were used to collect quantitative data reported within the manuscript. Click here for file
