# Supplementary material for: Knowledge and perception of pulmonary tuberculosis in pastoral communities in the middle and Lower Awash Valley of Afar region, Ethiopia
Source: BMC Public Health. 2010 Apr 12;10:187. doi: 10.1186/1471-2458-10-187 (PMC2867998; doi:10.1186/1471-2458-10-187)
Supplement: Additional file 1 — Table S1. Association of respondents' socio-demographic characteristics with respondents' knowledge of symptoms, mode of transmission, choice of effective treatment and preventive methods of PTB. Association of respondents socio-demographic characteristics and four domains of the level of knowledge about PTB is investigated using logistic regression. Odds ratio and 95% CI are reported within the body of the table. [file 1471-2458-10-187-S1.DOC]

Table 6. Association of respondents’ socio-demographic characteristics with respondents’ knowledge of symptoms, mode of transmission, choice of effective treatment and preventive methods of PTB

| Characteristic | Symptom | | Mode of Transmission | | Choice of treatment | | Preventive method | |
| --- | --- | --- | --- | --- | --- | --- | --- | --- |
|  | COR (95% CI) | AOR (95% CI) | COR (95% CI) | AOR (95% CI) | COR (95% CI) | AOR (95% CI) | COR (95% CI) | AOR (95% CI) |
| District  Dubti  Amibara | Reference  0.84 (0.63-1.13) | Reference  0.97(0.70- 1.32 | Reference  0.99 (0.71- 1.38) | Reference  0.87(0.61- 1.24) | Reference  1.02 (0.65-1.61) | Reference  0.99 (0.62-1.59) | Reference  0.99 (0.71- 1.38) | Reference  1.06 (0.75- 1.51) |
| Gender**:**  Female  Male | Reference  0.69 (0.51- 0.93 | Reference  0.65 (0.47- 0 .89) | Reference  1.42 (1.00- 2.01) | Reference  1.37(0.96-1.96) | Reference  2.17(1.37- 3.45) | Reference  2.21(1.37- 3.57) | Reference  0.81(0.58-1.14) | Reference  0.84 (0.59-1.19) |
| Age (years):  18-29  30-44  45-59  60+ | Reference  0.95 (0.66-1.36)  1.35 (0.87-2.09)  0.93 (0.45-1.92) | Reference  0.93 (0.63-1.38)  1.23 (0.76- 1.98)  0.99 (0.45- 2.14) | Reference  0.78 (0.52- 1.17)  0.93 (0.56- 1.54)  1.40 (0.66- 2.95) | Reference  0.79 (0.51- 1.22)  1.02 (0.60-1.73)  1.41 (0.65-3.06) | Reference  0.71(0.40-1.26)  1.11(0.52- 2.39)  0.73 (0.25-2 .11) | Reference  0.72 (0.39-1.30)  1.15 (0.52- 2.56)  0.62 (0.21-1.85) | Reference  0.98 (0.66- 1.46)  1.12 (0.68- 1.86)  1.04 (0.46- 2.36) | Reference  0.96 (0.63- 1.47)  0.97 (0.57- 1.67)  0.91 (0.38- 2.16) |
| Educational status  Illiterate  Literate | Reference  1.29 (0.76- 2.18) | Reference  1.49 (0.84- 2.67) | Reference  1.06 (0.58- 1.92) | Reference  0.97 (0.52- 1.81) | Reference  1.18 (0.49- 2.83) | Reference  0.83 (0.33-2.11) | Reference  1.00 (0.56 - 1.81) | Reference  1.02 (0.54 - 1.92) |
| Occupation :  Pastoralist  Agro-pastoralist | Reference  3.70 (2.68- 5.12) | Reference  3.66 (2.63- 5.08) | Reference  0.39 (0.25- 0.59) | Reference  0.38 (0.25- 0.58) | Reference  0.56 (0.35- 0.89) | Reference  0.55 (0.34- 0 .89) | Reference  3.78 (2.42- 5.89) | Reference  3.79 (2.42-5.93) |

COR= crude odd ratio, AOR =adjusted odd ratio
