# Supplementary material for: Knowledge and perception of pulmonary tuberculosis in pastoral communities in the middle and Lower Awash Valley of Afar region, Ethiopia
Source: BMC Public Health. 2010 Apr 12;10:187. doi: 10.1186/1471-2458-10-187 (PMC2867998; doi:10.1186/1471-2458-10-187)
Supplement: Additional file 2 — Questionnaires administered in the study. The questionnaire has all the questions that were used to collect quantitative data reported within the manuscript. [file 1471-2458-10-187-S2.DOC]

Questionnaire for assessment of awareness about pulmonary tuberculosis (PTB) in rural pastoral communities of Afar Region

**Part I. Socio- demographic characteristics of the respondents**

1. Name/ code of the respondent_________________________________

District **____________________________**Kebele**__________________** Village**________________________________-** House No**._____________**

1. Sex **:** 1= Male 2= Female
2. Age (Year): ____________

4. Ethnicity**:** 1= Afar 2= other specify ________________________________

5. Religion 1= Muslim 2= other specify _________________________________

6. Marital status 1= Married 2= single 3 = divorced 4= widowed

7. Educational status: 1= illiterate 2= read & write 3 = only read 4= primary (1-8) 5= secondary (9-12) 6= other (specify)_______________________-

8. Occupation: 1= Nomadic pastoralist 2= Agro-pastoralist 3= Merchant

4= Daily laborer 5= other (specify) ____________________________

9. Duration of residence in the area/village _______________ (years)

### Part II. Questionnaire about Knowledge of PTB

1. Have you ever heard about the disease called PTB (**Labadore** in Afar language) ?

1= Yes 2= No

2. If yes, from whom ?

1= From friend (s) 2= From health workers 3= From media (radio, TV poster etc) 4 = from patient 5= I/my family member had got the disease

6= Other(specify)___________________________

3. What do you think is the cause of PTB? **(do not read the alternatives but circle it if the respondent will mention it)**

1= Bacteria/germ 2= cold air 3= shortage of food 4= smoking, chewing , drinking 5= hot climate 6= sun light 7= dust 8= any other ________________________________

4. What are some of the common symptoms of PTB? (**do not read the alternatives but circle it if the respondent will mention it)**

1= Cough for 3 or more weeks 2 = sputum with blood 3= weight loss

4= loss of appetite 5= Fever and sweat at night 6= chest pain

7= any other________________________________________________

5. Do you think that the disease can transmits from patient to other person?

1= Yes 2= No 3= Do not know

6. If yes, how the disease can be transmitted from patient to other person ? **(do not read the alternatives but circle it if the respondent will mention it)**

1= through cough, sneeze & breath 2= through sharing materials for drink

3= sharing materials for feeding 4. contact with patients (body, cloth , sweat etc) 5=Any other --------------------------------

7. Do you think that the transmission of PTB is preventable?

1= yes 2= no 3= do not know

8. If yes , what measures do you think to be taken to prevent transmission of PTB from patient to another person? **do not read the alternatives but circle it if the respondent will mention it)**

1= avoid sharing cups with a patient 2= Do not cough/ sneeze at other people

3= Do not spit everywhere 4= use separate room for patient 4= early treatment 5= do not know 6= any other_____________________________

9. Does PTB have a treatment?

1= Yes 2= No 3= Do not know

10. What do you think is the effective treatment for PTB?

1= Traditional medicine 2 = Modern drug 3= both 4= do not know

11. If traditional medicine, what type of treatment is it?

1= Medicinal plants 2= Food (specify) __________________

3= any other _____________________

12. Have you/your family ever sick from PTB?

1= Yes 2= No 3= do not remember

13. If yes, measures taken to prevent the transmission of the disease to other family members?

1= separate room 2= separate drinking materials 3= early treatment 4= any other ___________________________________________________________

**Part III. Assessment of Perception of Communities about Public Health Importance of PTB**

1. . Is PTB a major health problem in this area?

1= Yes 2 = No 3= Rare 4=Don’t know

2. If yes, since when the disease is becoming a health problem in this area?

1= since recent years 2 = since an ancient time 3= Do not know 4= any other_____________________________________________________________

3. If since recent years, what factors do you think to contribute to its expansion ? (**do not read the alternatives but circle it if the respondent will mention it)**

1= HIV/AIDS 2= Poverty 3= climate change 4= Increasing of habits like smoking, chewing , drinking

5= any other _____________________________________________

4. In this area, PTB mostly attacks

1= children under 5 years 2= children 5-15 years 3= adults 4= very old people (over 60 years) 5= all 6= do not know

7= any other ______________________________

5. If one of the above age groups (e.g children under 5 years or old people), why do you think that PTB mostly attacks this age group? ________________________________________________________________________

6. PTB mostly attacks :

1 = men 2= women 3= both 4= do not know 5= any other _______________________________

7. If men or why ? _______________________________________________

**Part iv. Assessment of Community Perception about Risk factors for Eexposure to PTB and Disease Development**

1. Which of the followings do you think are risk factors for exposure/acquiring PTB in this area ?

a) Cups sharing habit among several peoples regardless of his/ her healthy status :

1= yes 2 = no 3 = do not know

b) Living with a PTB patient in a single room house (Afar house) :

1= yes 2 = no 3= do not know

c) Habit of chewing khat in group regardless of the healthy status of a person :

1= yes 2 = no 3 = do not know

d) Any other ____________________________________________________________

2. In your area, what type of persons mostly develops PTB ?

a) Those who frequently chewing khat/smoking :

1 = yes 2= no 3= do not know

b) Those who have problem of shortage food :

1= yes 2= no 3= do not know

c) Those who suffer from other diseases like HIV/AIDS:

1= yes 2= no 3 = do not know

d) Those who are under stress because of various reasons like family death, family conflict etc : 1= yes 2 = no 3 = do not know

e) any other
